# 9,9-Dimethyl-12-(4-nitro­phen­yl)-9,10-dihydro-12*H*-benzo[*a*]xanthen-11(8*H*)-one

**DOI:** 10.1107/S1600536809048570

**Published:** 2009-11-21

**Authors:** De-Ling Li, Li-Hong Wang

**Affiliations:** a Department of Chemistry, Tangshan Normal College, Tangshan 063000, People’s Republic of China

## Abstract

In the mol­ecular structure of the title compound, C_25_H_21_NO_4_, the pyran ring adopts a flattened boat conformation, while the cyclo­hexenone ring is in an envelope conformation. The 4-nitro­phenyl ring is almost perpendicular to the pyran ring [dihedral angle = 89.39 (1)°]. In the crystal, mol­ecules are connected by inter­molecular C—H⋯O hydrogen bonds.

## Related literature

For the biological activity of xanthenes and benzoxanthenes, see: Lambert *et al.* (1997 ▶); Poupelin *et al.* (1978 ▶); Ion *et al.* (1998 ▶); Saint-Ruf *et al.* (1975 ▶).
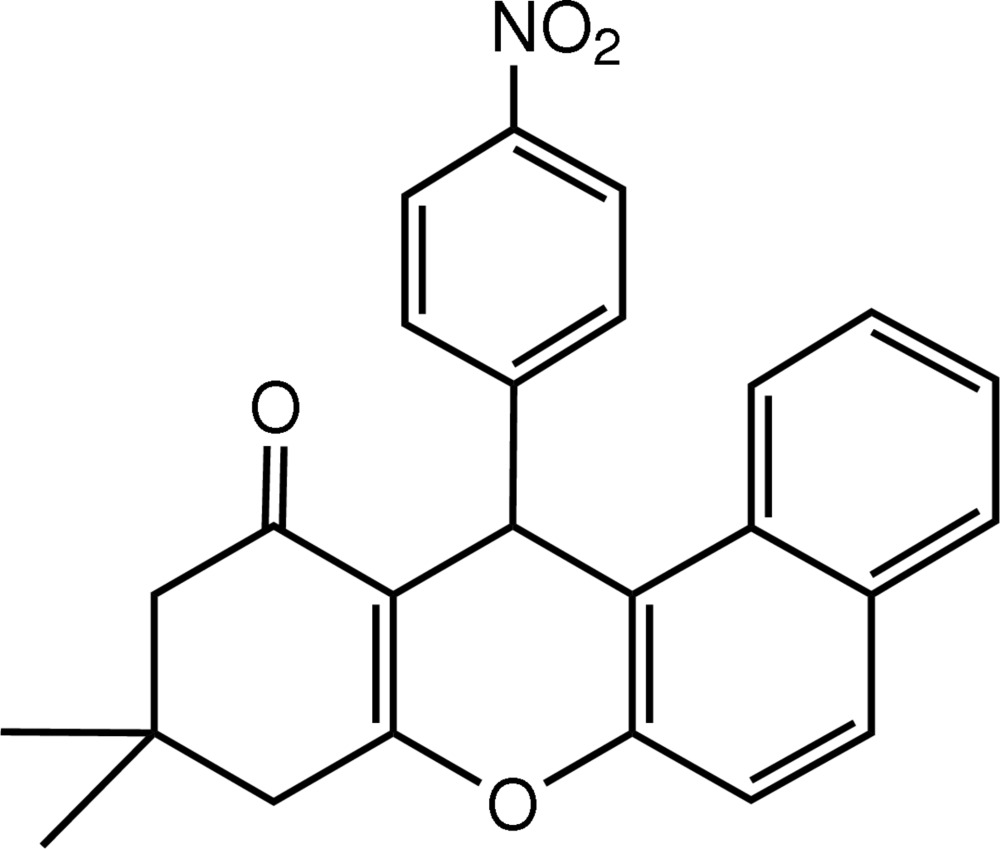


## Experimental

### 

#### Crystal data

C_25_H_21_NO_4_
*M* *_r_* = 399.43
Monoclinic, 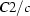

*a* = 24.178 (5) Å
*b* = 11.078 (2) Å
*c* = 17.481 (4) Å
β = 119.78 (3)°
*V* = 4063.9 (19) Å^3^
*Z* = 8
Mo *K*α radiation
μ = 0.09 mm^−1^
*T* = 113 K
0.20 × 0.18 × 0.10 mm

#### Data collection

Rigaku Saturn CCD area-detector diffractometer
Absorption correction: multi-scan (*CrystalClear*; Rigaku/MSC, 2005 ▶) *T*_min_ = 0.983, *T*_max_ = 0.999
14653 measured reflections
4007 independent reflections
3106 reflections with *I* > 2σ(*I*)
*R*_int_ = 0.044

#### Refinement

*R*[*F* ^2^ > 2σ(*F* ^2^)] = 0.055
*wR*(*F* ^2^) = 0.153
*S* = 1.03
4007 reflections
274 parameters
H-atom parameters constrained
Δρ_max_ = 0.29 e Å^−3^
Δρ_min_ = −0.26 e Å^−3^

### 

Data collection: *CrystalClear* (Rigaku/MSC, 2005 ▶); cell refinement: *CrystalClear*; data reduction: *CrystalClear*; program(s) used to solve structure: *SHELXS97* (Sheldrick, 2008 ▶); program(s) used to refine structure: *SHELXL97* (Sheldrick, 2008 ▶); molecular graphics: *SHELXTL* (Sheldrick, 2008 ▶); software used to prepare material for publication: *SHELXTL*.

## Supplementary Material

Crystal structure: contains datablocks I, global. DOI: 10.1107/S1600536809048570/bh2257sup1.cif

Structure factors: contains datablocks I. DOI: 10.1107/S1600536809048570/bh2257Isup2.hkl

Additional supplementary materials:  crystallographic information; 3D view; checkCIF report

## Figures and Tables

**Table 1:** Hydrogen-bond geometry (Å, °)

*D*—H⋯*A*	*D*—H	H⋯*A*	*D*⋯*A*	*D*—H⋯*A*
C2—H2⋯O4^i^	0.95	2.42	3.323 (3)	159
C6—H6⋯O2^ii^	0.95	2.45	3.384 (3)	168
C18—H18*B*⋯O2^iii^	0.98	2.43	3.355 (2)	158

Symmetry codes: (i) 
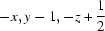
; (ii) 
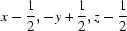
; (iii) 
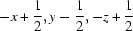
.

**Fig. 1.**
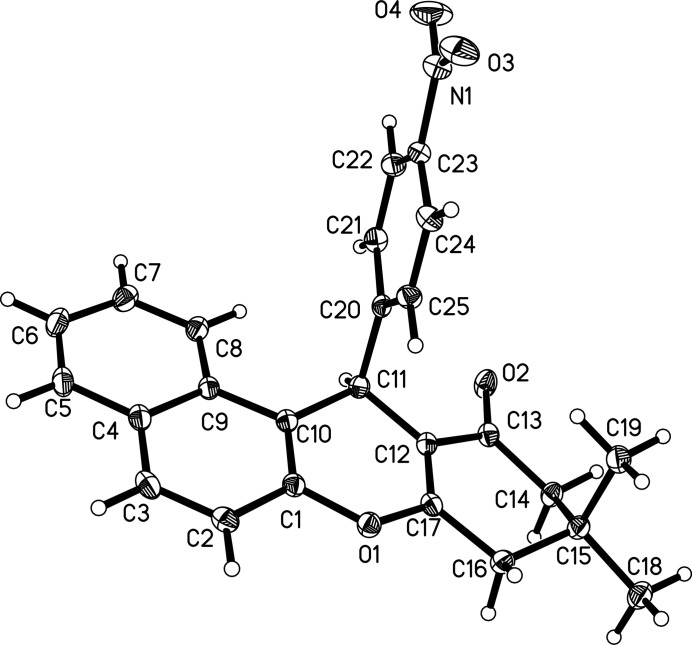
A view of the molecular structure of the title compound, showing the atom-numbering scheme. Displacement ellipsoids are drawn at the 30% probability level.

**Fig. 2.**
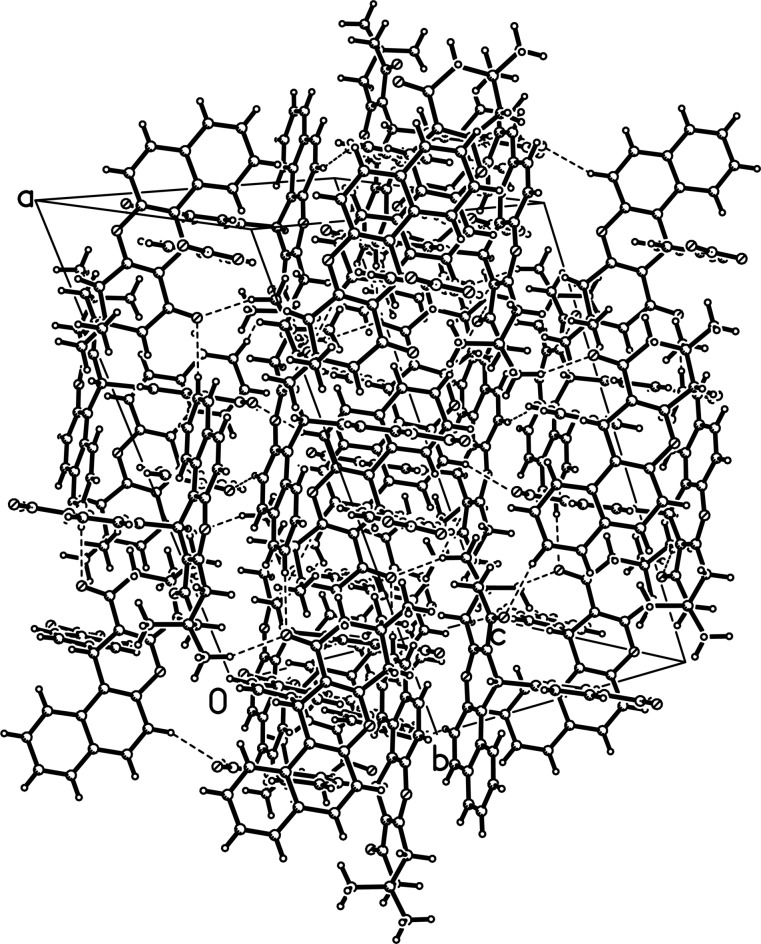
The packing, showing hydrogen-bond interactions as dashed lines; H atoms are shown as small spheres of arbitrary radii.

## supplementary crystallographic information

### Comment

Xanthenes and benzoxanthenes are biologically important drug intermediates. They are cited as active oxygen heterocycles possessing anti-inflammatory (Poupelin *et al.*, 1978) and antiviral (Lambert *et al.*, 1997) activity. These compounds are also utilized as antagonists for paralyzing action of zoxazolamine (Saint-Ruf *et al.*, 1975) and in photodynamic therapy (Ion *et al.*, 1998). We report herein the crystal structure of the title compound, which belongs to this class of compounds.

The pyran ring of the title molecule (Fig. 1) adopts a flattened boat conformation. The cyclohexenone ring is in an envelope conformation with atom C15 at the flap. The 4-nitrophenyl ring and the planar part of the pyran ring (C1/C10/C12/C17) are nearly perpendicular to each other, with a dihedral angle of 89.39 (1)°. In the crystal, the molecules are connected by C—H···O hydrogen bonds (Fig. 2).

### Experimental

To a mixture of 2-naphthol (1.0 mmol), benzaldehyde (1.0 mmol), and 5,5-dimethylcyclohexane-1,3-dione (1.1 mmol) was added strontium trifluoromethanesulfonate (0.1 mmol) in 1,2-dichloroethane (2 ml). The mixture was stirred at 353 K for 5 h. The progress of the reaction was monitored by TLC. After completion of the reaction, water was added and the product was extracted with ethyl acetate (3×10 ml). The organic layer was dried (MgSO_4_) and evaporated, and the crude product was purified by flash chromatography on silica gel. Pure product crystallized slowly at room temperature in ethanol. A single-crystal was obtained by slow evaporation of a solution in ethanol.

### Refinement

H atoms were included in the refinement in the riding and rotation model approximation, with C—H = 0.95–1.00 Å and *U*_iso_ (H) = 1.2 *U*_eq_(Carrier atom). For the methyl H atoms, *U*_iso_(H) = 1.5*U*_eq_(C).

### Crystal data

**Table:**

C_25_H_21_NO_4_	*F*(000) = 1680
*M**_r_* = 399.43	*D*_x_ = 1.306 Mg m^−^^3^
Monoclinic, *C*2/*c*	Mo *K*α radiation, λ = 0.71073 Å
Hall symbol: -C 2yc	Cell parameters from 4847 reflections
*a* = 24.178 (5) Å	θ = 2.1–27.9°
*b* = 11.078 (2) Å	µ = 0.09 mm^−^^1^
*c* = 17.481 (4) Å	*T* = 113 K
β = 119.78 (3)°	Prism, yellow
*V* = 4063.9 (19) Å^3^	0.20 × 0.18 × 0.10 mm
*Z* = 8	

### Data collection

**Table:**

Rigaku Saturn CCD area-detector diffractometer	4007 independent reflections
Radiation source: rotating anode	3106 reflections with *I* > 2σ(*I*)
confocal	*R*_int_ = 0.044
Detector resolution: 7.31 pixels mm^-1^	θ_max_ = 26.0°, θ_min_ = 2.1°
ω and φ scans	*h* = −29→26
Absorption correction: multi-scan (*CrystalClear*; Rigaku/MSC, 2005)	*k* = −13→12
*T*_min_ = 0.983, *T*_max_ = 0.999	*l* = −18→21
14653 measured reflections	

### Refinement

**Table:**

Refinement on *F*^2^	Secondary atom site location: difference Fourier map
Least-squares matrix: full	Hydrogen site location: inferred from neighbouring sites
*R*[*F*^2^ > 2σ(*F*^2^)] = 0.055	H-atom parameters constrained
*wR*(*F*^2^) = 0.153	*w* = 1/[σ^2^(*F*_o_^2^) + (0.0882*P*)^2^] where *P* = (*F*_o_^2^ + 2*F*_c_^2^)/3
*S* = 1.03	(Δ/σ)_max_ < 0.001
4007 reflections	Δρ_max_ = 0.29 e Å^−^^3^
274 parameters	Δρ_min_ = −0.26 e Å^−^^3^
0 restraints	Extinction correction: *SHELXS97* (Sheldrick, 2008), Fc^*^=kFc[1+0.001xFc^2^λ^3^/sin(2θ)]^-1/4^
0 constraints	Extinction coefficient: 0.0043 (6)
Primary atom site location: structure-invariant direct methods	

### Fractional atomic coordinates and isotropic or equivalent isotropic displacement parameters (Å^2^)

**Table:**

	*x*	*y*	*z*	*U*_iso_*/*U*_eq_	
O1	0.07668 (6)	−0.09846 (11)	0.23338 (8)	0.0274 (3)	
O2	0.23273 (6)	0.17081 (12)	0.24963 (8)	0.0360 (4)	
O3	0.06051 (8)	0.52066 (14)	0.42025 (11)	0.0504 (5)	
O4	0.08357 (10)	0.64016 (14)	0.34280 (11)	0.0625 (5)	
N1	0.07521 (9)	0.53889 (15)	0.36366 (11)	0.0385 (5)	
C1	0.02775 (9)	−0.03873 (17)	0.16146 (11)	0.0247 (4)	
C2	−0.03123 (9)	−0.09880 (17)	0.12366 (12)	0.0295 (5)	
H2	−0.0351	−0.1738	0.1469	0.035*	
C3	−0.08261 (9)	−0.04833 (18)	0.05346 (12)	0.0321 (5)	
H3	−0.1227	−0.0880	0.0283	0.038*	
C4	−0.07735 (9)	0.06226 (18)	0.01731 (12)	0.0292 (5)	
C5	−0.13056 (10)	0.1170 (2)	−0.05572 (13)	0.0365 (5)	
H5	−0.1710	0.0787	−0.0805	0.044*	
C6	−0.12508 (10)	0.2230 (2)	−0.09105 (13)	0.0397 (6)	
H6	−0.1615	0.2591	−0.1388	0.048*	
C7	−0.06498 (10)	0.2782 (2)	−0.05608 (12)	0.0370 (5)	
H7	−0.0607	0.3505	−0.0819	0.044*	
C8	−0.01239 (9)	0.22896 (17)	0.01486 (11)	0.0295 (5)	
H8	0.0277	0.2683	0.0378	0.035*	
C9	−0.01689 (8)	0.12084 (17)	0.05423 (11)	0.0243 (4)	
C10	0.03670 (8)	0.06891 (16)	0.13055 (11)	0.0230 (4)	
C11	0.10058 (8)	0.13313 (16)	0.17849 (11)	0.0233 (4)	
H11	0.1119	0.1628	0.1340	0.028*	
C12	0.15111 (8)	0.04569 (16)	0.23939 (11)	0.0237 (4)	
C13	0.21800 (9)	0.07594 (17)	0.27101 (11)	0.0263 (4)	
C14	0.26749 (9)	−0.01679 (17)	0.32757 (12)	0.0291 (5)	
H14A	0.3089	0.0244	0.3635	0.035*	
H14B	0.2727	−0.0752	0.2887	0.035*	
C15	0.25060 (9)	−0.08591 (16)	0.38902 (11)	0.0278 (4)	
C16	0.18478 (9)	−0.14376 (17)	0.33158 (12)	0.0276 (4)	
H16A	0.1893	−0.2140	0.3001	0.033*	
H16B	0.1686	−0.1736	0.3701	0.033*	
C17	0.13742 (9)	−0.05788 (16)	0.26568 (11)	0.0241 (4)	
C18	0.30005 (10)	−0.18485 (18)	0.43696 (13)	0.0361 (5)	
H18A	0.3425	−0.1484	0.4702	0.054*	
H18B	0.2995	−0.2421	0.3938	0.054*	
H18C	0.2899	−0.2275	0.4776	0.054*	
C19	0.24992 (10)	0.00103 (19)	0.45700 (12)	0.0350 (5)	
H19A	0.2913	0.0418	0.4892	0.052*	
H19B	0.2418	−0.0445	0.4985	0.052*	
H19C	0.2163	0.0613	0.4266	0.052*	
C20	0.09628 (8)	0.24126 (16)	0.22956 (11)	0.0232 (4)	
C21	0.10351 (9)	0.35856 (17)	0.20785 (12)	0.0285 (4)	
H21	0.1129	0.3716	0.1617	0.034*	
C22	0.09739 (10)	0.45691 (18)	0.25190 (12)	0.0323 (5)	
H22	0.1023	0.5369	0.2366	0.039*	
C23	0.08393 (9)	0.43547 (16)	0.31859 (12)	0.0276 (4)	
C24	0.07792 (9)	0.32046 (17)	0.34403 (12)	0.0293 (5)	
H24	0.0698	0.3081	0.3914	0.035*	
C25	0.08398 (9)	0.22379 (17)	0.29877 (11)	0.0270 (4)	
H25	0.0797	0.1440	0.3151	0.032*	

### Atomic displacement parameters (Å^2^)

**Table:**

	*U*^11^	*U*^22^	*U*^33^	*U*^12^	*U*^13^	*U*^23^
O1	0.0245 (7)	0.0279 (7)	0.0284 (7)	−0.0022 (5)	0.0120 (6)	0.0018 (5)
O2	0.0243 (7)	0.0399 (9)	0.0376 (8)	−0.0038 (6)	0.0108 (6)	0.0098 (6)
O3	0.0587 (11)	0.0439 (10)	0.0597 (10)	−0.0063 (8)	0.0378 (9)	−0.0174 (8)
O4	0.1033 (16)	0.0236 (9)	0.0641 (11)	0.0058 (9)	0.0444 (11)	−0.0003 (8)
N1	0.0419 (11)	0.0297 (10)	0.0373 (10)	0.0006 (8)	0.0146 (8)	−0.0073 (8)
C1	0.0236 (10)	0.0268 (10)	0.0230 (9)	0.0009 (7)	0.0110 (7)	−0.0040 (7)
C2	0.0292 (11)	0.0291 (10)	0.0342 (10)	−0.0044 (8)	0.0189 (8)	−0.0061 (8)
C3	0.0240 (11)	0.0381 (12)	0.0352 (11)	−0.0068 (8)	0.0155 (8)	−0.0149 (9)
C4	0.0229 (10)	0.0370 (11)	0.0268 (10)	0.0015 (8)	0.0116 (8)	−0.0099 (8)
C5	0.0223 (10)	0.0499 (14)	0.0295 (10)	0.0035 (9)	0.0070 (8)	−0.0131 (9)
C6	0.0316 (12)	0.0508 (14)	0.0271 (10)	0.0158 (10)	0.0073 (8)	−0.0033 (10)
C7	0.0407 (13)	0.0418 (13)	0.0253 (10)	0.0114 (10)	0.0140 (9)	0.0018 (9)
C8	0.0288 (11)	0.0347 (11)	0.0238 (9)	0.0054 (8)	0.0123 (8)	−0.0012 (8)
C9	0.0198 (10)	0.0301 (10)	0.0230 (9)	0.0025 (8)	0.0106 (7)	−0.0062 (7)
C10	0.0214 (10)	0.0268 (10)	0.0224 (9)	0.0006 (7)	0.0120 (7)	−0.0037 (7)
C11	0.0208 (9)	0.0258 (10)	0.0237 (9)	0.0000 (7)	0.0113 (7)	0.0006 (7)
C12	0.0216 (10)	0.0270 (10)	0.0213 (9)	0.0005 (7)	0.0097 (7)	−0.0005 (7)
C13	0.0243 (10)	0.0308 (11)	0.0232 (9)	−0.0001 (8)	0.0113 (8)	−0.0012 (8)
C14	0.0213 (10)	0.0326 (11)	0.0305 (10)	0.0031 (8)	0.0107 (8)	0.0007 (8)
C15	0.0262 (11)	0.0280 (10)	0.0263 (10)	0.0051 (8)	0.0108 (8)	0.0006 (8)
C16	0.0293 (11)	0.0257 (10)	0.0270 (9)	0.0017 (8)	0.0134 (8)	0.0018 (8)
C17	0.0210 (10)	0.0269 (10)	0.0238 (9)	−0.0024 (7)	0.0106 (7)	−0.0046 (7)
C18	0.0330 (12)	0.0374 (12)	0.0334 (10)	0.0082 (9)	0.0130 (8)	0.0046 (9)
C19	0.0327 (11)	0.0378 (12)	0.0289 (10)	0.0044 (9)	0.0112 (8)	−0.0031 (9)
C20	0.0174 (9)	0.0247 (10)	0.0235 (9)	−0.0005 (7)	0.0069 (7)	0.0009 (7)
C21	0.0286 (10)	0.0297 (10)	0.0271 (9)	−0.0025 (8)	0.0137 (8)	0.0025 (8)
C22	0.0361 (12)	0.0223 (10)	0.0314 (10)	−0.0022 (8)	0.0115 (9)	0.0028 (8)
C23	0.0263 (10)	0.0225 (10)	0.0276 (10)	0.0003 (8)	0.0084 (8)	−0.0042 (8)
C24	0.0317 (11)	0.0301 (11)	0.0279 (10)	−0.0041 (8)	0.0162 (8)	−0.0051 (8)
C25	0.0301 (10)	0.0232 (10)	0.0276 (10)	−0.0033 (8)	0.0143 (8)	0.0010 (8)

### Geometric parameters (Å, °)

**Table:**

O1—C17	1.362 (2)	C12—C13	1.465 (3)
O1—C1	1.393 (2)	C13—C14	1.513 (3)
O2—C13	1.226 (2)	C14—C15	1.531 (3)
O3—N1	1.223 (2)	C14—H14A	0.9900
O4—N1	1.227 (2)	C14—H14B	0.9900
N1—C23	1.464 (2)	C15—C18	1.529 (2)
C1—C10	1.370 (2)	C15—C19	1.536 (3)
C1—C2	1.406 (3)	C15—C16	1.538 (3)
C2—C3	1.359 (3)	C16—C17	1.494 (3)
C2—H2	0.9500	C16—H16A	0.9900
C3—C4	1.413 (3)	C16—H16B	0.9900
C3—H3	0.9500	C18—H18A	0.9800
C4—C5	1.422 (3)	C18—H18B	0.9800
C4—C9	1.427 (3)	C18—H18C	0.9800
C5—C6	1.364 (3)	C19—H19A	0.9800
C5—H5	0.9500	C19—H19B	0.9800
C6—C7	1.406 (3)	C19—H19C	0.9800
C6—H6	0.9500	C20—C21	1.389 (2)
C7—C8	1.373 (3)	C20—C25	1.395 (3)
C7—H7	0.9500	C21—C22	1.384 (3)
C8—C9	1.412 (3)	C21—H21	0.9500
C8—H8	0.9500	C22—C23	1.378 (3)
C9—C10	1.440 (2)	C22—H22	0.9500
C10—C11	1.519 (2)	C23—C24	1.381 (3)
C11—C12	1.507 (2)	C24—C25	1.382 (2)
C11—C20	1.528 (2)	C24—H24	0.9500
C11—H11	1.0000	C25—H25	0.9500
C12—C17	1.337 (2)		
			
C17—O1—C1	118.36 (14)	C13—C14—H14B	108.9
O3—N1—O4	123.25 (17)	C15—C14—H14B	108.9
O3—N1—C23	118.92 (17)	H14A—C14—H14B	107.7
O4—N1—C23	117.82 (18)	C18—C15—C14	109.56 (16)
C10—C1—O1	122.67 (16)	C18—C15—C19	109.35 (15)
C10—C1—C2	123.27 (17)	C14—C15—C19	109.78 (15)
O1—C1—C2	114.06 (16)	C18—C15—C16	109.46 (15)
C3—C2—C1	119.24 (19)	C14—C15—C16	107.49 (15)
C3—C2—H2	120.4	C19—C15—C16	111.18 (16)
C1—C2—H2	120.4	C17—C16—C15	112.73 (15)
C2—C3—C4	121.03 (18)	C17—C16—H16A	109.0
C2—C3—H3	119.5	C15—C16—H16A	109.0
C4—C3—H3	119.5	C17—C16—H16B	109.0
C3—C4—C5	121.93 (19)	C15—C16—H16B	109.0
C3—C4—C9	119.44 (17)	H16A—C16—H16B	107.8
C5—C4—C9	118.62 (19)	C12—C17—O1	122.74 (16)
C6—C5—C4	121.7 (2)	C12—C17—C16	125.83 (17)
C6—C5—H5	119.1	O1—C17—C16	111.42 (16)
C4—C5—H5	119.1	C15—C18—H18A	109.5
C5—C6—C7	119.32 (19)	C15—C18—H18B	109.5
C5—C6—H6	120.3	H18A—C18—H18B	109.5
C7—C6—H6	120.3	C15—C18—H18C	109.5
C8—C7—C6	120.9 (2)	H18A—C18—H18C	109.5
C8—C7—H7	119.6	H18B—C18—H18C	109.5
C6—C7—H7	119.6	C15—C19—H19A	109.5
C7—C8—C9	121.07 (19)	C15—C19—H19B	109.5
C7—C8—H8	119.5	H19A—C19—H19B	109.5
C9—C8—H8	119.5	C15—C19—H19C	109.5
C8—C9—C4	118.34 (17)	H19A—C19—H19C	109.5
C8—C9—C10	122.60 (17)	H19B—C19—H19C	109.5
C4—C9—C10	119.05 (18)	C21—C20—C25	118.47 (16)
C1—C10—C9	117.88 (17)	C21—C20—C11	121.29 (16)
C1—C10—C11	120.43 (16)	C25—C20—C11	120.23 (16)
C9—C10—C11	121.63 (16)	C22—C21—C20	121.44 (17)
C12—C11—C10	109.51 (15)	C22—C21—H21	119.3
C12—C11—C20	110.94 (14)	C20—C21—H21	119.3
C10—C11—C20	110.11 (14)	C23—C22—C21	118.07 (17)
C12—C11—H11	108.7	C23—C22—H22	121.0
C10—C11—H11	108.7	C21—C22—H22	121.0
C20—C11—H11	108.7	C22—C23—C24	122.59 (17)
C17—C12—C13	118.81 (16)	C22—C23—N1	118.57 (17)
C17—C12—C11	122.81 (17)	C24—C23—N1	118.85 (17)
C13—C12—C11	118.38 (16)	C23—C24—C25	118.20 (17)
O2—C13—C12	120.95 (17)	C23—C24—H24	120.9
O2—C13—C14	121.74 (17)	C25—C24—H24	120.9
C12—C13—C14	117.25 (16)	C24—C25—C20	121.19 (17)
C13—C14—C15	113.47 (16)	C24—C25—H25	119.4
C13—C14—H14A	108.9	C20—C25—H25	119.4
C15—C14—H14A	108.9		
			
C17—O1—C1—C10	−10.1 (2)	C17—C12—C13—C14	3.6 (2)
C17—O1—C1—C2	169.76 (15)	C11—C12—C13—C14	−176.40 (15)
C10—C1—C2—C3	−0.7 (3)	O2—C13—C14—C15	146.74 (17)
O1—C1—C2—C3	179.44 (15)	C12—C13—C14—C15	−35.8 (2)
C1—C2—C3—C4	1.0 (3)	C13—C14—C15—C18	174.83 (15)
C2—C3—C4—C5	−179.58 (17)	C13—C14—C15—C19	−65.1 (2)
C2—C3—C4—C9	1.0 (3)	C13—C14—C15—C16	56.0 (2)
C3—C4—C5—C6	−179.19 (18)	C18—C15—C16—C17	−165.35 (15)
C9—C4—C5—C6	0.2 (3)	C14—C15—C16—C17	−46.4 (2)
C4—C5—C6—C7	1.8 (3)	C19—C15—C16—C17	73.74 (19)
C5—C6—C7—C8	−2.2 (3)	C13—C12—C17—O1	−173.81 (15)
C6—C7—C8—C9	0.7 (3)	C11—C12—C17—O1	6.2 (3)
C7—C8—C9—C4	1.3 (3)	C13—C12—C17—C16	5.4 (3)
C7—C8—C9—C10	−177.74 (17)	C11—C12—C17—C16	−174.52 (16)
C3—C4—C9—C8	177.69 (16)	C1—O1—C17—C12	9.3 (2)
C5—C4—C9—C8	−1.7 (3)	C1—O1—C17—C16	−170.01 (14)
C3—C4—C9—C10	−3.3 (3)	C15—C16—C17—C12	17.8 (3)
C5—C4—C9—C10	177.33 (16)	C15—C16—C17—O1	−162.89 (14)
O1—C1—C10—C9	178.31 (14)	C12—C11—C20—C21	123.51 (18)
C2—C1—C10—C9	−1.6 (3)	C10—C11—C20—C21	−115.09 (19)
O1—C1—C10—C11	−4.4 (3)	C12—C11—C20—C25	−57.4 (2)
C2—C1—C10—C11	175.71 (16)	C10—C11—C20—C25	64.0 (2)
C8—C9—C10—C1	−177.50 (16)	C25—C20—C21—C22	−1.4 (3)
C4—C9—C10—C1	3.5 (3)	C11—C20—C21—C22	177.65 (17)
C8—C9—C10—C11	5.3 (3)	C20—C21—C22—C23	0.1 (3)
C4—C9—C10—C11	−173.76 (15)	C21—C22—C23—C24	1.5 (3)
C1—C10—C11—C12	17.4 (2)	C21—C22—C23—N1	−177.96 (17)
C9—C10—C11—C12	−165.45 (15)	O3—N1—C23—C22	176.83 (18)
C1—C10—C11—C20	−104.88 (19)	O4—N1—C23—C22	−3.7 (3)
C9—C10—C11—C20	72.3 (2)	O3—N1—C23—C24	−2.7 (3)
C10—C11—C12—C17	−18.7 (2)	O4—N1—C23—C24	176.80 (19)
C20—C11—C12—C17	103.10 (19)	C22—C23—C24—C25	−1.8 (3)
C10—C11—C12—C13	161.39 (15)	N1—C23—C24—C25	177.68 (17)
C20—C11—C12—C13	−76.85 (19)	C23—C24—C25—C20	0.4 (3)
C17—C12—C13—O2	−178.88 (17)	C21—C20—C25—C24	1.1 (3)
C11—C12—C13—O2	1.1 (3)	C11—C20—C25—C24	−177.95 (16)

### Hydrogen-bond geometry (Å, °)

**Table:**

*D*—H···*A*	*D*—H	H···*A*	*D*···*A*	*D*—H···*A*
C2—H2···O4^i^	0.95	2.42	3.323 (3)	159
C6—H6···O2^ii^	0.95	2.45	3.384 (3)	168
C18—H18B···O2^iii^	0.98	2.43	3.355 (2)	158

Symmetry codes: (i) −*x*, *y*−1, −*z*+1/2; (ii) *x*−1/2, −*y*+1/2, *z*−1/2; (iii) −*x*+1/2, *y*−1/2, −*z*+1/2.
